# The effectiveness of community dance in people with cancer: a mixed-methods systematic review and meta-analysis

**DOI:** 10.1093/heapro/daad077

**Published:** 2023-08-03

**Authors:** Eimear Nelson, Dervla Kelly, Orfhlaith Ni Bhriain, Fran Garry, Amanda M Clifford, Joanna M Allardyce

**Affiliations:** ULCaN, Health Research Institute, University of Limerick, Limerick, Ireland; School of Medicine, Health Research Institute, University of Limerick, Limerick, Ireland; IWAMD—Irish World Academy of Music and Dance, ULCaN, Health Research Institute, University of Limerick, Limerick, Ireland; IWAMD—Irish World Academy of Music and Dance, ULCaN, Health Research Institute, University of Limerick, Limerick, Ireland; School of Allied Health, Ageing Research Centre, Health Research Institute, University of Limerick, Limerick, Ireland; School of Allied Health, ULCaN, Health Research Institute, University of Limerick, Limerick, Ireland

**Keywords:** community-based intervention, physical activity, cancer, community health promotion, dance intervention

## Abstract

There is a need for both feasible and enjoyable physical activity programmes for people on a cancer journey. Emerging evidence suggests that dance can have a positive effect on health and well-being in this cohort. We aimed to synthesize the quantitative and qualitative literature exploring the effectiveness and impact of community dance interventions in people with all types and stages of cancer. A systematic search was performed following Preferred Reporting Items for Systematic Reviews and Meta-Analyses guidelines in Pubmed, EMBASE, Medline Ovid, CINAHL and PEDro databases. Quantitative and qualitative data were extracted and synthesized using a convergent segregated approach. The numeric data were analysed using descriptive statistics, narrative synthesis and meta-analysis where possible. The qualitative data were analysed using thematic analysis. The Downs and Black critical appraisal tool and the Critical Appraisal Skills Programme were used to assess the quality of the quantitative and qualitative literature, respectively. Eighteen studies were included in this mixed-methods review with seven trials included in the meta-analysis. Statistically significant improvements were found in favour of community dance for functional capacity, fatigue, quality-of-life and depression in comparison to no intervention. Evidence suggests dance is a safe and feasible form of physical activity both during and after cancer treatment. Participants reported good social support, education regarding physical activity and local access as key facilitators to participation. We concluded that dance is a feasible and enjoyable intervention for many people with various forms of cancer. Community dance programmes can improve both physical and psychological outcomes in people on a cancer journey.

**Contribution to Health Promotion**

- Physical activity can help physical and mental well-being.
- Dance can positively impact day-to-day tasks, fatigue levels and mental health in people along a cancer journey.
- Community dance classes are a safe and feasible exercise for people with all types and stages of cancer.

## BACKGROUND

Cancer prevalence is increasing due to improvements in life expectancy, cancer screening and detection (Hulvat, 2020). In Ireland, cancer survivorship has doubled in the last decade (National Cancer Registry Ireland, 2022). Despite advances in detection, treatment and 5-year survival rates, there remains a need for better management of the physical and psychological cancer-related issues of people with cancer (Ward *et al.*, 2020).

Psychological issues including anxiety, depression, sexual dysfunction, sleep disturbance, fatigue and cognitive impairments experienced by people at all stages of a cancer journey are well documented (Stein *et al.*, 2008; Cramer *et al.*, 2012; Sundarsean et al., 2015). All of which can negatively affect a person’s quality-of-life (QoL). Fear of progression and recurrence of cancer frequently lead to increased distress in both cancer patients and survivors (Dinkel and Herschbach, 2017). Physically, cancer survivors can present with functional impairments (Clifford *et al.*, 2018).

Physical activity (PA), defined as any movement that increases energy expenditure, can elicit a multitude of health benefits through a range of activity forms (World Health Organisation, 2022). PA has been shown to reduce fatigue, improve physical fitness and improve health-related QoL in people with cancer (Cramp *et al.*, 2012; Mishra *et al.*, 2012; Furmaniak *et al.*, 2016; Zhu *et al.*, 2016). Up to 70% of cancer survivors are not sufficiently active due to barriers such as a lack of motivation, high fatigue levels or individual time constraints (Eng *et al.*, 2018). Cancer survivors have reported enjoyment as a key facilitator to PA (Höh *et al.*, 2018). Thus, it is essential to explore enjoyable feasible forms of exercise to facilitate the uptake and participation of PA.

Dance has been found to be a safe, inexpensive and enjoyable method of PA in older adults (Roberson *et al.*, 2014; Franco *et al.*, 2020). Dance is a broad term characterized as moving a person’s body rhythmically to music (Fong Yan *et al.*, 2018). Community dance encompasses a range of styles but the key universal element is a pattern of movement to music performed in a group setting. Previous reviews have synthesized the literature to examine the effect of community dance and dance movement therapy (DMT) in people with cancer. Improvements were reported in health-related QoL and physical functioning after community dance and DMT interventions (Rudolph *et al.*, 2018). Dance was also found to improve psychological and physical outcomes such as QoL, depression, self-esteem and functional capacity in women with breast cancer (Bradt *et al.*, 2015; Boing *et al.*, 2017). To date, no published mixed-methods review has evaluated the impact of community dance on people with cancer at different stages in their cancer journey. This review aims to examine the quantitative and qualitative literature to assess the effectiveness of, and opinion on community dance in people with cancer to guide recommendations for the design of future dance interventions in an Irish community setting.

Moreover, to the author’s knowledge, no studies have explored the exercise prescription for dance interventions in people living with cancer to date. A recent systematic review explored exercise dosage in resistance training for breast cancer patients and concluded low volume resistance training may result in higher improvements in muscle strength than higher-volume training (Lopez *et al.*, 2021). It is important to review the dosage of interventions in a dance setting to ensure participants attain benefits whilst also ensuring the prescription is realistic and achievable.

Therefore, the aims of this systematic review are:

1. To evaluate the effect of community dance interventions on the psychological and physical outcomes in people with all types and stages of cancer, using both qualitative and quantitative data.
2. To provide a synopsis of dance prescription in the existing literature.

## METHODS

The Preferred Reporting Items for Systematic Reviews and Meta-Analyses (PRISMA) checklist was complied with in this review (Moher *et al.*, 2009). Additionally, the Joanna Briggs Institute Manual for Evidence Synthesis informed the integration of qualitative and quantitative data (Lizarondo *et al.*, 2022).

### Inclusion criteria

*Study design*: All studies with primary data; randomized controlled trials (RCTs), non-RCTs, clinical trials and qualitative studies published in English that evaluated the effectiveness of dance programmes on people living with cancer. There was no time restriction applied to ensure inclusion of all available literature. *Population:* Studies that included people with any type and any stage of cancer who were undergoing active or inactive treatment, or cancer survivors, that explored physical and psychological outcomes collected quantitatively or qualitatively. *Intervention*: Studies that investigated any type of dance intervention. *Comparison*: Studies that compared dance to another intervention or to a control group.

### Exclusion criteria

Studies that included DMT were excluded. The term dance is used as an umbrella term for both DMT and community dance therefore, herein it is important to distinguish the two. DMT is a type of psychotherapy administered by a professionally trained therapist, linking the movement and emotion of participants to elicit intellectual, psychological and physical improvements (ADMT, 2022, EADMT, 2022). The requisite of a trained specialist renders this intervention unfeasible in some locations. Therefore, our inclusion criteria focussed solely on community dance. In addition, papers that prescribed dance together with another intervention, other than usual care, were excluded.

### Literature search

**Fig. 1:**
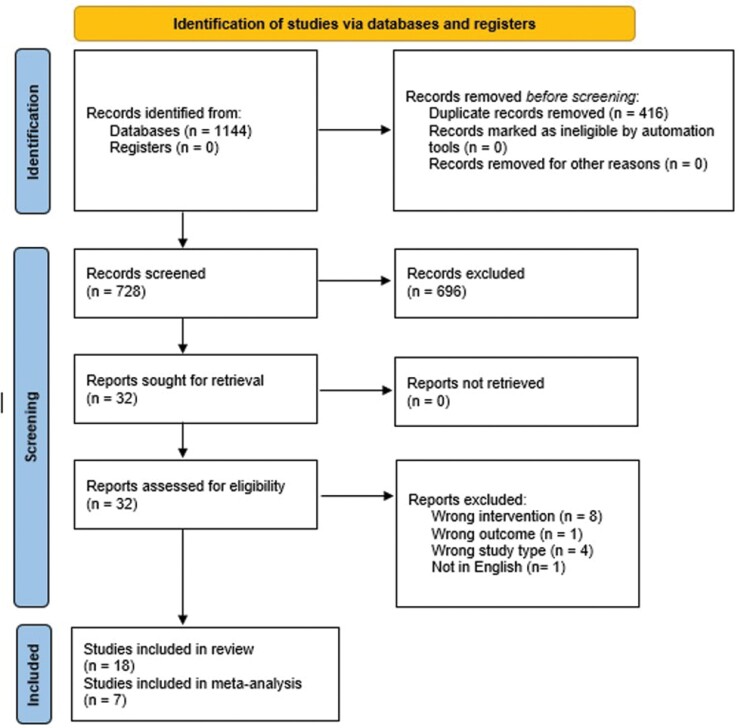
PRISMA flowchart.

An electronic literature search was carried out in October 2022 on five databases: Pubmed, EMBASE, Medline Ovid, CINAHL and Pedro using the MESH search terms (cancer OR tumour OR tumor OR carcinoma OR lymphoma OR leukemia OR neoplasm) and (active treatment OR inactive treatment OR radiotherapy OR chemotherapy OR survivor) and (dance OR dance therapy OR dancing OR movement to dance, NOT dance movement therapy OR dmt). Findings of the search were exported to Endnote and duplicates were removed. The subsequent papers were then exported to Rayyan QCRI systematic review software and were screened by title and abstract based on predefined inclusion criteria, by one screener (E.N.). Potential studies were next read in full to determine eligibility. Any uncertainties were independently screened by a second reviewer (A.C.) and 100% agreement was reached. The reference lists of all included papers were screened by title to find any additional papers. A summary of the search process can be seen in Figure 1 PRISMA flowchart.

### Quality assessment

The Downs and Black quality appraisal tool was used to assess the quality of the quantitative clinical trials (Downs and Black, 1998). There is a maximum score of 28 points, with a higher score indicating better quality. Previous studies assigned a classification based on their score: excellent (26–28), good (20–25), fair (15–19) and poor (≤14) (Hooper *et al.*, 2008; Silverman *et al.*, 2012).

The Critical Appraisal Skills Programme (CASP, 2022) was used to appraise the qualitative studies to address validity and study relevance (Singh, 2013).

### Data extraction

Data were extracted by a single reviewer (E.N.) under key headings; author, type of study, participants (type of cancer, stage of cancer, mean age), sample size, dropouts, exercise prescription (under frequency, intensity, time and type framework) and description of the intervention. Quantitative results were extracted into a Microsoft Excel document under the following headings: pre-intervention and post-intervention results, mean difference, standard deviation and other statistical interactions. Qualitative data were extracted using line-by-line coding by two reviewers (E.N., A.C.) independently and key themes were formed in relation to the review question.

### Data analysis

The included studies were categorized into dosage levels to allow some comparison regarding dosage; high—achieving >150 min of activity per week, moderate—achieving 100–150 min per week and low—achieving <100 min per week.

Data from each study were compared under each outcome of interest. A meta-analysis was performed, using the Review Manager 5 software, when at least three studies shared a common outcome. The data were inputted as continuous, and the inverse variance was selected as the statistical method. Either the fixed effect analysis model or the random effects analysis model was utilized depending on the homogeneity of the studies. The mean difference or standard mean difference was utilized for the comparison of studies with the same outcome measure or different outcome measures, respectively. All meta-analysis used the 95% confidence interval (CI).

Thematic synthesis was carried out on the qualitative data using our outcomes of interest. A convergent segregated approach was then taken to synthesize and integrate the qualitative and quantitative data (Hong *et al.*, 2017).

## RESULTS

### Study selection

Literature search yielded 1144 results. 416 duplicates were removed and 728 screened by title and abstract. Thirty-two papers were read in full and 18 met the inclusion criteria (Figure 1). No additional papers were found from reference lists. Two authors were contacted to obtain further data. Jenkins and Wakeling (Jenkins and Wakeling, 2020) provided a booklet with further qualitative data.

### Study characteristics

A total of 18 papers were included in this review, consisting of 16 unique population groups (Table 1). Szalai *et al*. (Szalai et al., 2015, 2017) utilized the same population group to assess different outcomes, as did Carminatti *et al*. (Carminatti *et al.*, 2019) and Boing *et al*. (Boing *et al.*, 2018). These study pairs will be grouped as one to avoid overrepresentation of study demographics. Ten studies compared a dance intervention to another arm (Supplementary Material 2.1). Six studies had no control group.

**Table 1:** Study characteristics

Type of Study	Data type	Critical appraisal score	Author Country	Sample Size (analysed) (I:C)	Participants		Mean age (±SD)	
					Type of cancer	Stage of treatment	I	C
RCT	Quantitative	26 (D&B) Excellent	He *et al.* (2022) China	176 (88:88)	Breast Stage I or III	Post surgery or under treatment	47.99 (5.54)	48.32 (10.00)
RCT	Quantitative	25 (D&B) Good	Leite *et al.* (2021) Brazil	52 (18:18:16)	Breast Stage 0–III	Undergoing adjuvant hormone therapy	53 (10)	58 (11)
RCT	Quantitative and Qualitative—exit interviews	23 (D&B) Good	Pisu *et al.* (2017) USA	31 couples (15:16)	Any: endometrial, ovarian, breast and colorectal	Post treatment (>3 months)	57.9	57.9
RCT	Quantitative	19 (D&B) Fair	Kaltsatou *et al.* (2011) Greece	27 (14:13)	Breast	Post surgery, post treatment (>3 months)	56.6 (4.2)	57.1 (4.1)
RCT	Quantitative	19 (D&B) Fair	Karathanou *et al.* (2020) Greece	300 (150:150)	Any: breast, prostate, lung and colon Stage I or II	Under or post treatment	58.12 (8.32)	59.28 (9.11)
RCT	Quantitative and Qualitative—exit interviews	18 (D&B) Fair	Soltero *et al.* (2022) USA	20 (10:10)	Breast Stage 0–III	Post treatment	49.6 (6.22)	53.2 (9.30)
Non-randomised controlled trial	Quantitative	23 (D&B) Good	Sturm *et al.* (2014) Germany	40 (20:20)	Any: breast, ovarian and gastrointestinal	Under treatment	49	50.5
Non-randomised controlled trial	Quantitative	21 (D&B) Good	Szalai *et al.* (2015)*** Hungary	114 (55:59)	Any	Post treatment	48.87 (1.197)	51.31 (1.440)
Non-randomised controlled trial	Quantitative	19 (D&B) Fair	Carminatti *et al.* (2019)** Brazil	19 (8:11)	Breast Stage I–III	Under or post treatment	54.55 (8.29)	54.55 (8.29)
Non-randomised controlled trial	Quantitative	19 (D&B) Fair	Boing *et al.* (2018)** Brazil	19 (8:11)	Breast Stage I–III	Under or post treatment	54.55 (8.29)	54.55 (8.29)
Non-randomised controlled trial	Quantitative and Qualitative—informal reports	19 (D&B) Fair	Hiansdt *et al.* (2021) Brazil	21 (11:10)	Breast Stage I–III	Under or post treatment	55.7 (7.3)	54.8 (9.6)
One-group pretest–posttest design	Quantitative	15 (D&B) Fair	Loo *et al.* (2019) Hawaii	11	Breast Stage I–III	Post treatment (6–60 months)	63 (10.2)	—
One-group pretest–posttest design	Quantitative	12 (D&B) Poor	Thieser *et al.* (2021) Germany	66	Any	Under or post treatment	unclear	
One-group pretest–posttest design	Quantitative	11 (D&B) Poor	Schmidt *et al.* (2018) Germany	9 patients 4 partners	Any: breast, colorectal, prostate and melanoma	Under or post treatment	65* 56–75>	—
Clinical report	Qualitative—informal reports	7 (D&B) Poor	Molinaro *et al.* (1986) USA	37	Breast	Post surgery	55.5* 30–81	—
Qualitative, descriptive study	Qualitative—focus groups	8 (CASP)	Butler *et al.* (2016) New Zealand	8	Any	Under or post treatment	65* 50–80	—
Qualitative, descriptive study	Qualitative Semi structured interviews	8 (CASP)	Szalai *et al.* (2017)*** Hungary	51	Any	Post treatment	48.51 (1.256)	—
Qualitative, descriptive study	Qualitative—surveys	3 (CASP)	Jenkins and Wakeling (2020) UK	40	Any	unclear	57.5*	

Abbreviations: CASP, Critical Appraisal Skills Programme; D&B, Downs and Black Critical Appraisal Tool; I:C, intervention: control; SD, standard deviation; *mean not provided, thus calculated from median of range, **paper with same population—Carminatti and Boing, ***paper with same population—Szalai and Szalai.

### Participants

In total 971 participants, from 8 countries, were included across 16 groups. The sample size varied from 8 to 300. Participants were between 25 and 90 years old, most commonly in their 50s. Eight studies solely included people with breast cancer, skewing gender distribution towards females. Seven studies investigated the effect of dance both during and post treatment, two looked at during only, and six post treatment only. Three studies invited participants to bring a partner or friend.

### Exercise prescription

The frequency of the interventions varied from one to five times per week, sessions lasted between 30 and 90 min. The intensity was omitted by most studies, however, when provided it was measured by maximum heart rate. Duration of the studies varied from 5 to 52 weeks, resulting in a total dosage ranging between 10 and 81 h (Supplementary Material 2.1). three studies achieved >150 min of activity per week, 6 studies achieved 100–150 min per week and 4 achieved <100 min per week. Three studies did not include enough information.

### Dance genre

In many cases, the genre was not specified. The commonalities were that the interventions could be classed as community-based classes incorporating exercise to music, dance and choreography. All were focussed on group participation. Some were based on specific traditions such as ballet and folk dance, others were creating movement and participating in community classes. They ranged from solo to partner to circle/line dances. In all cases, classes were community-based and sought to create cohesion and individual empowerment.

### Quality appraisal

Quantitative studies scores ranged from 7 (poor) to 26 (excellent) on the Downs and Black critical appraisal tool. The average score was 18.3 points indicating fair quality overall. Two qualitative studies scored 8/10 on the CASP checklist and one scored 3/10 (Supplementary Material 1.2).

### Adherence

Adherence rates were reported in six studies and varied from 46 to 84% among participants during and post treatment stage (Sturm *et al.*, 2014; Pisu *et al.*, 2017; Boing *et al.*, 2018; Carminatti *et al.*, 2019; Loo *et al.*, 2019; Hiansdt *et al.*, 2021; Soltero *et al.*, 2022).

### Adverse effects

Details of adverse effects resulting from the intervention were reported in five studies. Sturm *et al*. reported three Grade 1 muscle aches and one case of aggravation of pre-existing knee pain after dance intervention in participants undergoing treatment (Sturm *et al.*, 2014). The remaining four reported no adverse effects in patients both undergoing or post treatment (Schmidt *et al.*, 2018; Hiansdt *et al.*, 2021; Leite *et al.*, 2021; He *et al.*, 2022).

### Dropouts

Attrition was observed in the interventions due to the fluctuating side effects of treatment. Seven of the 16 studies reported dropouts ranging between 7 and 41% (Pisu *et al.*, 2017; Boing *et al.*, 2018; Schmidt *et al.*, 2018; Carminatti *et al.*, 2019; Loo *et al.*, 2019; Leite *et al.*, 2021; He *et al.*, 2022; Soltero *et al.*, 2022). Three studies provided the reasons for dropouts; physical effects of treatment, lack of finances for transport costs, lack of time, loss of interest and other disease/metastasis (Boing *et al.*, 2018; Carminatti *et al.*, 2019; Leite *et al.*, 2021; He *et al.*, 2022).

### Meta-analysis

**Fig. 2:**
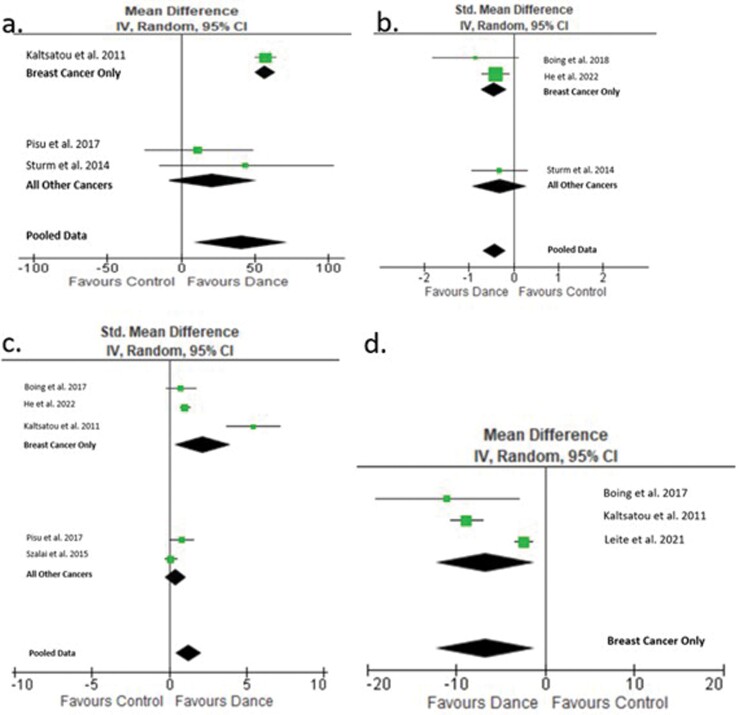
Meta-analysis results. Forest plots generated from meta-analysis for (a) functional capacity. Mean difference (95% CI) of effect of dance intervention on functional capacity (*N* = 3 6MWT) compared to no intervention. Data collected from three studies, *N* = 96. (b) Fatigue. Standard mean difference (95% CI) of effect of dance intervention on fatigue (*N* = 1 Piper Fatigue Scale, *N* = 1 Brief Fatigue Inventory and *N* = 1 Functional Assessment of Chronic Illness Therapy: Fatigue) compared to no intervention. Data collected from three studies, *N* = 229. (c) QoL. Standard mean difference (95% CI) of effect of dance intervention on QoL [*N* = 1 The European Organization for Research and Treatment of Cancer—Functional scale, *N* = 1 Functional assessment of cancer therapy—breast (FACT-B): total score, *N* = 1 Life Satisfaction Inventory, *N* = 1 Short Form 12—Mental Component Summary and *N* = 1 The European Organization for Research and Treatment of Cancer QoL Core Questionnaire] compared to no intervention. Data collected from 5 studies, *N* = 359. (d) Depression. Mean difference (95% CI) of effect of dance intervention on depression (*N* = 3 Becks Depression Inventory) compared to no intervention. Data collected from three studies, *N* = 80.

Eight studies included similar outcomes (functional capacity, fatigue, QoL and depression) allowing comparison through further statistical analysis (Carminatti *et al.*, 2019; Boing *et al.*, 2018; Kaltsatou *et al.*, 2011; Sturm *et al.*, 2014; Pisu *et al.*, 2017; Leite *et al.*, 2021; He *et al.*, 2022). Carminatti *et al*. and Boing *et al*. were not compared to prevent duplication of data. A total of seven studies were included in our meta-analysis (Figure 2).

### Quantitative data

This study included all types of cancer to improve applicability of the findings, however, it must be recognized that half of the included studies looked at breast cancer alone. To ensure clarity when presenting results, the meta-analysis has been subdivided into breast cancer only and any type of cancer.

### Primary outcomes

#### Functional capacity

Three studies were included in our meta-analysis to assess the effect of dance on functional capacity using the 6 Minute Walk Test (6MWT). Dance resulted in significant improvements in functional capacity (40.66 points, 95% CI 8.95–72.36; *p* = 0.01, *I*^2^ = 66%) (Kaltsatou *et al.*, 2011; Sturm *et al.*, 2014; Pisu *et al.*, 2017) (Table 3.1 in Supplementary Material). One additional study reported a correlation between increase in 6MWT score and the more weeks of dance classes engaged in (*p* = 0.000) (Thieser *et al.*, 2021).

#### Fatigue

Four studies explored the effect of dance on fatigue levels. Three studies utilizing different outcome measures were included in the meta-analysis (Sturm *et al.*, 2014; Boing *et al.*, 2018; Carminatti *et al.*, 2019; He *et al.*, 2022). A significant effect was found in favour of dance in comparison to a control for fatigue (−0.42 points, 95% CI −0.69 to −0.16; *p* = 0.002, *I*^2^ = 0%) (Table 3.2 in Supplementary Material). The one study not included in the meta-analysis due to lack of control group found non-significant improvements in fatigue after a hula intervention in all three fatigue-related outcome measures (Loo *et al.*, 2019).

#### QoL

Eight studies evaluated the effect of a dance intervention on QoL using various outcome measures. Five studies were included in a meta-analysis and found a significant improvement in QoL in favour of dance, compared to a control group (1.27 points, 95% CI 0.40–2.14; *p* = 0.004, *I*^2^ = 91%) (Table 3.3 in Supplementary Material) (Kaltsatou *et al.*, 2011; Szalai *et al.*, 2015; Pisu *et al.*, 2017; Boing *et al.*, 2018; Carminatti *et al.*, 2019; He *et al.*, 2022). The studies that could not be included in the meta-analysis reported non-statistically significant improvements in QoL following dance, (Loo *et al.*, 2019) *p* = 0.15; (Schmidt *et al.*, 2018) *p* > 0.05; Sturm *et al.*, 2014 *p* > 0.05).

#### Psychological

Three outcomes were investigated under the heading psychological: depression, stress and anxiety (Table 3.4 in Supplementary Material).

Five studies explored the effect of dance on depression. Three studies comparing dance to a control group were included in a meta-analysis and found statistically significant improvements in Becks Depression Inventory (−6.73 points, 95% CI −12.28 to −1.18; *p* = 0.02, *I*^2^ = 95%) (Kaltsatou *et al.*, 2011; Boing *et al.*, 2018; Leite *et al.*, 2021). One study had a third-arm Pilates group and found improvements in both belly dance and Pilates groups compared to the control group, but no significant between the group differences post-intervention (Leite *et al.*, 2021). One study did not find significant improvements in depression post-hula intervention (*p* = 0.17) (Loo *et al.*, 2019). Only one study individually reported statistically significant differences between dance and control group post-intervention (He *et al.*, 2022).

One study reported stress significantly improved post-Greek dance intervention in the dance group and reported a significant deterioration in the control group (Karathanou *et al.*, 2020).

Karathanou *et al*. (Karathanou *et al.*, 2020) showed significant improvements in anxiety in both groups post-Greek dance. Conversely, Loo *et al*. (Loo *et al.*, 2019) showed non-statistical improvements in hula dance group both post-intervention and at 104-weeks follow-up (*p* = 0.09).

#### Relationships

Three studies evaluated the effect of a dance intervention on interpersonal relationships. Szalai *et al*. demonstrated a significant improvement in favour of the dance group post-belly dance (*p* = 0.000) (Szalai *et al.*, 2015). However, two studies did not find significant improvements after a ballroom and hula dance intervention, respectively (Pisu *et al.*, 2017; Loo *et al.*, 2019) (Table 3.5 in Supplementary Material).

#### Body image

Carminatti *et al* found significant improvements in body stigma (*p* = 0.017) and transparency (*p* = 0.021) (Carminatti *et al.*, 2019). The same population was investigated by Boing *et al*. which demonstrated significant improvements in body image (*p* = 0.037) (Boing *et al.*, 2018). In contrast, Thieser *et al*. did not find a significant influence of dance on body image scores (*p* = 0.156) (Thieser *et al.*, 2021) (Table 3.6 in Supplementary Material).

#### PA levels

Two studies found significant improvements in PA levels after the dance intervention. Pisu *et al*. (Pisu *et al.*, 2017) reported significant between group differences in favour of ballroom dance with a partner (*p* < 0.05) and Loo *et al*. reported significant improvements at a 52-week follow-up for hula dance (*p* < 0.05) (Loo *et al.*, 2019). In contrast, Boing *et al*. reported a non-significant reduction in activity levels (*p* = 0.088) (Boing *et al.*, 2018). Furthermore, Soltero *et al*. (Soltero *et al.*, 2022) found no statistical differences on PA when comparing dance with tai chi (Table 3.2 in Supplementary Material).

### Qualitative data

Qualitative data were captured through primary data and direct quotes from the participants (Butler *et al.*, 2016; Szalai *et al.*, 2017; Soltero *et al.*, 2022) and summary of patient perceptions (Molinaro *et al.*, 1986; Pisu *et al.*, 2017; Hiansdt *et al.*, 2021) (Table 1). One study provided direct quotes upon contacting the author (Jenkins and Wakeling, 2020).

#### Qualitative themes

Key issues identified were the importance of dance in providing social support and improving the body image of the participants.

### A welcoming community

Five studies discussed the importance of social support and comradery during the intervention. These studies highlighted the importance of group comradery (Butler *et al.*, 2016) and the desire to belong to a group and reap social benefits (Szalai *et al.*, 2017; Jenkins and Wakeling, 2020). Additionally, these groups allowed advice sharing (Molinaro *et al.*, 1986) and allowed participants and their partners to appreciate the time they spent together during the sessions (Pisu *et al.*, 2017).

### Body confidence

The effect of dance on the participants’ confidence and body image was widely noted through feedback from the participants. Some qualitative evidence depicts negative body thoughts prior to exercise (Szalai *et al.*, 2017) and demonstrated that body confidence grew after the dance intervention (Butler *et al.*, 2016). Participants reported feelings of reconnection and a new appreciation for their bodies (Szalai *et al.*, 2017; Jenkins and Wakeling, 2020).

## DISCUSSION

The results of this review are based on robust and standardized synthesis and analysis of the available literature on dance in people with cancer. Our meta-analysis showed significant improvements in physical functioning and health-related QoL in various dance genres, similar to a previous review that noted improvements in these outcomes post-ballroom dance intervention (Rudolph *et al.*, 2018). Taken together with current evidence that dance, regardless of genre, can improve cardiovascular endurance and muscle strength in older adults (Hwang and Braun, 2015; Rodrigues-Krause *et al.*, 2018), can prevent functional loss regarding balance, gait and flexibility (Koch *et al.*, 2019), and have positive effects on the psychological, QoL and motor skills in various health conditions (Koch *et al.*, 2019), it is likely that community dance classes can be beneficial to people with cancer.

This review found statistically significant improvements in functional capacity and fatigue. These are important findings, people with cancer report a declining physical function as a debilitating side effect of treatment (Derks *et al.*, 2016). Similarly, fatigue is reported in up to 99% of cancer patients (O’Higgins *et al.*, 2018). Despite the high incidence rate, only four studies in our review measured fatigue. The findings of this review suggest dance genre may influence the effect on fatigue as both belly dance and hula dance showed no improvements in fatigue. Research demonstrated aerobic exercise can improve fatigue levels (Cramp and Byron-Daniel, 2012). These dances are not aerobically centred which may explain these results. Further investigation into the effect of dance genre on fatigue is needed to understand it is potential to improve this debilitating symptom. Importantly, reduced health-related QoL can be a predictor of mortality in people with cancer (Park *et al.*, 2016). Our meta-analysis showed significant improvement in QoL, following participation in a dance intervention. However notably, not all studies reported improvement. Interestingly, these studies also reported fluctuating attendance and higher dropout rates in comparison to studies that found significant improvements. Reduced adherence and enjoyment of the intervention may have contributed to a reduced effect on QoL.

We identified mixed results for the effect of community dance on stress, anxiety, relationships, PA and body image. Similar research on dance and other health conditions found no conclusive evidence for effect of community dance on anxiety, and body image due to the high heterogeneity of studies (Koch *et al.*, 2019). However, evidence supports the use of dance in improving depression. Given the emotional effects of cancer diagnosis and treatment, the role of dance in modifying psychological outcomes is important. Dance can influence brain functions which in turn can manifest improvements in psychological distress and anxiety (Brown *et al.*, 2006).

Regarding relationships, participants perceived an improvement in social support, evidenced in qualitative data but not quantitative. We hypothesis that the quantitative Dyadic Trust Scale may fail to find significant improvements despite the same cohort stating appreciation for their partner in exit interviews due to its design in 1980 making it outdated. Therefore, the qualitative data may be more appropriate to capture changes in social dynamics. Additionally, this review noted limited evidence of longer-term changes in PA levels, however, the inclusion of a partner appears to promote participation.

Body image can be improved through dance intervention. Participants expressed pride for their bodies qualitatively, which is mirrored through quantitative findings. Only specific subsections of body image outcome measures gave significant results indicating dance may only improve certain areas of body image. Notably, in this review, body image was investigated only in the belly dance genre. This genre encompasses spiritual elements greater than other dance types suggesting different genres may affect this.

Only two studies in this review compared dance to another physical intervention. In a 3-arm study, both Pilates and dance found improvements, albeit non-significant, in depression and self-esteem but no significant improvements between groups (Leite *et al.*, 2021). Recent systematic reviews highlighted the benefit of Pilates in women with breast cancer in functional capacity, range of motion, pain and fatigue (Costa Espindula *et al.*, 2017), however, did not find a difference between Pilates and other exercise programmes (Pinto-Carral *et al.*, 2018). Pilates programmes may be limited by lack of resources such as equipment or a trained instructor, therefore, alternative activities are warranted. Akin to Pilates, there was little difference when the dance was compared to tai chi (Soltero *et al.*, 2022). Further research is required to compare dance to other interventions.

### Cancer type

Community dance interventions may have a stronger effect on people with breast cancer, compared to other cancers? albeit both yielded positive benefits. To our knowledge, no study to date has compared cancer-type response to specific PA interventions. Exercise is beneficial across many cancer types on functional capacity, QoL and fatigue (Piraux *et al.*, 2020).

Several factors may influence the improvements seen in breast cancer. Dance as an intervention may be accepted more by females. Andeoli (Andeoli, 2019) suggests some males do not see it as masculine and may not fully engage. If there is a reduction in intensity or engagement, they may reap fewer benefits. Additionally, early detection can lead to reduced morbidity of treatment (Grimm *et al.*, 2022). Nationally, there has been increased screening and awareness leading to earlier diagnosis of breast cancer.

### Treatment status

This review included populations undergoing treatment and post treatment to ensure transferability of results across treatment status group. No significant differences were noted on our outcomes of interest. Previous research indicates that there are beneficial effects of exercise regardless of treatment status, however, it notes that the magnitude of these effects may differ for each outcome. Physical functioning, QoL and strength outcomes may be greater post treatment, whereas fatigue may be greater improved during treatment (Stout *et al.*, 2017). The multivariable nature of the included studies make it difficult to draw any definite conclusions regarding effect of treatment status on outcomes. However, it is promising that benefits were found indicating dance is a suitable form of PA across the cancer journey.

### Exercise prescription

Exercise is widely underprescribed and underachieved in the cancer cohort (Blanchard *et al.*, 2008; Bernardo *et al.*, 2010; Bao *et al.*, 2020). American College of Sports Medicine recommends the inclusion of moderate-intensity aerobic exercise at least three times per week, for 30 min minimum within a course of 8–12 weeks. Additionally, resistance training is recommended at least twice per week to improve cancer-related health outcomes such as fatigue and physical functioning (Campbell *et al.*, 2019).

Only three studies in this review met the recommended guidelines for exercise prescription (Kaltsatou *et al.*, 2011; Leite *et al.*, 2021; He *et al.*, 2022). However, our review found 45 min per week for 15 weeks demonstrated benefits in functional capacity and fatigue (Pisu *et al.*, 2017). Overall, increased duration of exercise prescription did not correlate linearly with greater improvements in our outcomes of interest. Low exercise dosage still shows promising results for dance, given that implementing a programme meeting the guidelines may not be realistic for sedentary patients. Emerging data even queries if lower dosage may be more appropriate in this cohort as these patients may have impairments in recovery response post treatment (Lopez *et al.*, 2021). Our quantitative findings support the need for low-intensity programmes initially to accustom patients to PA. Exercise prescription requires further investigation, taking into consideration the multivariable nature of study interventions.

All dance genres found benefits in some outcomes of interest except for hula dance. Although hula dance has been shown to improve core and back strength (Raorane *et al.*, 2019), our review did not show significant psychological or physical benefits. We hypothesize that given the repetitive motion, progression and variation are prevented (Kasper, 2019).

### Adherence and adverse effects

Adherence to the intervention was moderate. There was little correlation between dosage and adherence or attrition rates. Interestingly, two high-dose studies reported conflicting adherence rates, however, the populations differed by treatment status. Poorer adherence was reported in the group undergoing active treatment in comparison to the group under or post treatment. Similar findings were reported in people engaging in exercise during breast cancer treatment suggesting high-dosage interventions may affect adherence during active treatment (Courneya *et al.*, 2014). Both the quantitative and qualitative evidence cites the side effects of treatment as a barrier to exercise. In practice, allowances may have to be made for missed sessions in those undergoing treatment.

The number of dropouts was not correlated to the length of study, which illustrates that long-length studies are sustainable. Other reasons for dropouts such as location and time barriers are cited in the existing literature (Ormel *et al.*, 2018). Participation can be time consuming and costly, limiting engagement. On implementation of future classes, local classes or online classes should be considered to optimize adherence.

There were only mild side effects noted after a dance intervention in people undergoing treatment and no side effects noted in participants after treatment, suggesting that dance is a safe method of PA. Our findings are akin to other reviews on DMT, which found no adverse effects in patients with cancer (Bradt *et al.*, 2015). However, inadequate clarity reporting adverse effects means no definite conclusions can be drawn on patient safety.

### Study quality

In controlled trials, there was a universal lack of blinding of subjects due to the nature of the intervention. Blind assessors could have mitigated against assessor bias. Furthermore, the lack of adjustment for principal confounders and insufficiently powered studies were other possible shortfalls that contributed to reduced study quality. In the qualitative studies, the lack of detail on the role of the researcher and potential bias on data collection reduced quality. The Downs and Black tool for trials with no control group is not regularly recommended, however, it allowed for greater comparison across the studies.

### Strengths and limitations

Our inclusion of various study types provided a comprehensive synopsis of the literature to date. Similarly, qualitative evidence provided unique insights into participant opinion. The inclusion of different cancer stages and cancer types enhances the generalisability of our findings. Non-RCTs pose a challenge to the integrity of data and thus, reduce overall quality. RCTs are seen as the gold standard but can be less pragmatic making it difficult to translate findings to real-world scenarios (Østerås *et al.*, 2018).

One limitation of this review is the high heterogeneity of outcome measures used which affected the synthesis of results. Unfortunately, no further subgroup analysis could be performed due to the high heterogeneity of dance types, dosage and participants stage and type of cancer. Furthermore, the overrepresentation of breast cancer patients may bias the results as mentioned above. Other limitations include the small sample sizes and low-powered studies.

### Recommendations for research

No studies investigated functional capacity, QoL or fatigue qualitatively. In addition, there was little quantitative data on the effect of dance on stress, anxiety, relationships, body image and PA levels, preventing completion of a meta-analysis. Furthermore, only two studies compared dance to another physical intervention. Future research should investigate the effect of dance in comparison to other physical interventions to establish effectiveness.

Participants are rarely consulted despite evidence showing participation is improved when individual preferences, circumstances and experience are considered (Slade *et al.*, 2014). In future, a patient and public involvement panel would likely provide beneficial insights when designing programmes.

## CONCLUSION

Dance shows promising positive effects on physical and psychological outcomes in people with various types of cancer both during and after treatment. The optimum exercise prescription is yet to be established but as little as 45 min of dance per week has been shown to be feasible and effective. This research shows dance to be a safe intervention in people post treatment, however, more research is required in participants undergoing treatment to investigate the occurrence of adverse treatment-related effects. Additionally, the variety of intervention types and outcome measures demonstrate the need for further research to allow more definitive conclusions.

## Supplementary Material

daad077_suppl_Supplementary_Material

## References

[CIT0002] American Dance Therapy Association (ADTA). (2022) *American Dance Therapy Association*. https://www.adta.org/ (last accessed 30 November 2022).

[CIT0003] Andreoli, G. S. (2019) Dance and gender relations: a reflection on the interaction between male and female students in dance classes. Journal of Education and Social Policy, 6, 132–139.

[CIT0004] Bao, Y., Chen, S., Jiang, R., Li, Y., Chen, L., Li, F. et al. (2020) The physical activity of colorectal cancer survivors during chemotherapy. Supportive Care in Cancer, 28, 819–826. 31154534 10.1007/s00520-019-04873-3

[CIT0005] Bernardo, L., Abt, K., Ren, D. and Bender, C. (2010) Self-reported exercise during breast cancer treatment: results of a national survey. Cancer Nursing, 33, 304–309. 20467312 10.1097/NCC.0b013e3181cdce2c

[CIT0006] Blanchard, C. M., Courneya, K. S. and Stein, K.; American Cancer Society’s SCS-II. (2008) Cancer survivors’ adherence to lifestyle behaviour recommendations and associations with health-related quality of life: results from the American Cancer Society’s SCS-II. Journal of Clinical Oncology, 26, 2198–2204. 18445845 10.1200/JCO.2007.14.6217

[CIT0007] Boing, L., Fátima, B., Pereira, G. S., Sperandio, F. F., Moratelli, J., Cardoso, A. A. et al. (2018) Benefits of belly dance on quality of life, fatigue, and depressive symptoms in women with breast cancer – a pilot study of a non-randomised clinical trial. Journal of Bodywork & Movement Therapies, 22, 460–466. 29861250 10.1016/j.jbmt.2017.10.003

[CIT0008] Boing, L., Rafael, A. D., Braga, H., Sperandio, F. F., de Moraes, A. J. P. and Guimarães, A. C. A. (2017) Dance as treatment therapy in breast cancer patients – a systematic review. Brazilian Journal of Physical Activity and Health, 22, 319–331.

[CIT0009] Bradt, J., Shim, M. and Goodill, S. W. (2015) Dance/movement therapy for improving psychological and physical outcomes in cancer patients. Cochrane Database of Systematic Reviews. CD007103. doi:10.1002/14651858.CD007103.pub3. (15 July 2023, last accessed) 25565627 PMC7204197

[CIT0010] Brown, S., Martinez, M. J. and Parsons, L. M. (2006) The neural basis of human dance. Cerebral Cortex, 16, 1157–1167. 16221923 10.1093/cercor/bhj057

[CIT0011] Butler, M., Snook, B. and Buck, R. (2016) The transformative potential of community dance for people with cancer. Qualitative Health Research, 26, 1928–1938. 26386023 10.1177/1049732315602721

[CIT0012] Campbell, K. L., Winters-Stone, K. M., Wiskemann, J., May, A. M., Schwartz, A. L., Courneya, K. S. et al. (2019) Exercise guidelines for cancer survivors: consensus statement from international multidisciplinary roundtable. Medicine & Science in Sports & Exercise, 51, 2375–2390. 31626055 10.1249/MSS.0000000000002116 PMC8576825

[CIT0013] Carminatti, M., Boing, L., Leite, B., Flores Sperandio, F., Korpalski, T. et al. (2019) Effects of belly dancing on body image and self-esteem in women with breast cancer – pilot study. Revista Brasileira de Medicina do Esporte, 25, 464–468.

[CIT0014] Clifford, B. K., Mizrahi, D., Sandler, C. X., Barry, B. K., Simar, D., Wakefield, C. E. et al. (2018) Barriers and facilitators of exercise experienced by cancer survivors: a mixed methods systematic review. Support Cancer Care, 26, 685–700. 10.1007/s00520-017-3964-5 29185105

[CIT0015] Costa Espindula, R., Barbosa Nadas, G., Ines da Rosa, M., Foster, C., Cardoso de Araujo, F. and Grande, A. J. (2017) Pilates for breast cancer: a systematic review and meta-analysis. Revista da Associacao Medica Brasileira, 63, 1006–1012. 29451666 10.1590/1806-9282.63.11.1006

[CIT0016] Courneya, K. S., Segal, R. J., Gelmon, K., Mackey, J. R., Friedenreich, C. M., Yutaka, Y. et al. (2014) Predictors of adherence to different types and doses of supervised exercise during breast cancer chemotherapy. International Journal of Behavioral Nutrition and Physical Activity, 85. 10.1186/s12966-014-0085-0 PMC4110703 24997476

[CIT0017] Cramer, H., Lange, S., Klose, P., Paul, A. and Dobos, G. (2012) Can yoga improve fatigue in breast cancer patients? A systematic review. Acta Oncologia, 51, 559–560. 10.3109/0284186X.2011.637960 22136073

[CIT0018] Cramp, F. and Byron-Daniel, J. (2012) Exercise for the management of cancer-related fatigue in adults. Cochrane Database of Systematic Reviews, 11, CD006145. 23152233 10.1002/14651858.CD006145.pub3 PMC8480137

[CIT0019] Critical Appraisal Skills Programme. (2022) *CASP Qualitative Checklist*. https://casp-uk.net/casp-tools-checklists/ (last accessed 30 November 2022).

[CIT0021] Derks, M. G. M., de Glas, N. A., Bastiaannet, E., de Craen, A. J. M., Portielje, J. E. A., van de Velde, C. J. H. et al. (2016) Physical functioning in older patients with breast cancer: a prospective cohort study in the TEAM trial. The Oncologist, 21, 946–953. 27368882 10.1634/theoncologist.2016-0033 PMC4978563

[CIT0022] Dinkel, A. and Herschbach, P. (2017) Fear of progression in cancer patients and survivors. Recent Results in Cancer Research, 210, 13–33. 10.1007/978-3-319-64310-6_2 28924677

[CIT0023] Downs, S. H. and Black, N. (1998) The feasibility of creating a checklist for the assessment of the methodological quality both of randomised and non-randomised studies of health care intervention. Journal of Epidemiology & Community Health, 52, 377–384. 9764259 10.1136/jech.52.6.377 PMC1756728

[CIT0024] Eng, L., Pringle, D., Su, J., Shen, X., Mahler, M., Niu, C. et al. (2018) Patterns, perceptions, and perceived barriers to physical activity in adult cancer survivors. Supportive Care in Cancer, 26, 3755–3763. 29808379 10.1007/s00520-018-4239-5

[CIT0025] European Association Dance Movement Therapy (EADMT). (2022) *European Association Dance Movement Therapy*. https://www.eadmt.com/ (last accessed 30 November 2022).

[CIT0027] Fong Yan, A., Cobley, S., Chan, C., Pappas, E., Nicholson, L. L., Ward, R. E. et al. (2018) The effectiveness of dance interventions on physical health outcomes compared to other forms of physical activity: a systematic review and meta-analysis. Sports Medicine, 48, 933–951. 29270864 10.1007/s40279-017-0853-5

[CIT0028] Franco, M. R., Sherrington, C., Tiedemann, A., Pereira, L. S., Perracini, M. R., Faria, C. S. G. et al. (2020) Effect of senior dance (DanSE) on fall risk factors in older adults: a randomized controlled trial. Physical Therapy & Rehabilitation Journal, 100, 600–608. 31899491 10.1093/ptj/pzz187

[CIT0029] Furmaniak, A. C., Menig, M. and Markes, M. H. (2016) Exercise for women receiving adjuvant therapy for breast cancer. Cochrane Database of Systematic Reviews, 9, CD005001. 27650122 10.1002/14651858.CD005001.pub3 PMC6457768

[CIT0031] Grimm, L. J., Avery, C. S., Hendrick, E. and Baker, J. A. (2022) Benefits and risks of mammography screening in women ages 40 to 49 years. Journal Primary Care Community Health, 13, 1–6. 10.1177/21501327211058322 PMC8796062 35068237

[CIT0033] He, X., Ng, M. S. N., Choi, K. C. and So, W. K. W. (2022) Effects of a 16-week dance intervention on the symptom cluster of fatigue-sleep disturbance-depression and quality of life among patients with breast cancer undergoing adjuvant chemotherapy: a randomized controlled trial. International Journal of Nursing Studies, 133, 104317. 35850058 10.1016/j.ijnurstu.2022.104317

[CIT0034] Hiansdt, J. S., Boing, L., Sperandio, F. F., de Dem Fretta, T. Coutinho de Azevedo Guimares, A. (2021) The influence of 12-week dance intervention on sleep quality and pain among women with breast cancer – pilot study of a non-randomized clinical trial. Prevention and Rehabilitation, 26, 43–48. 10.1016/j.jbmt.2020.10.004 33992279

[CIT0035] Höh, J., Schmidt, T. and Hübner, J. (2018) Physical activity among cancer survivors – what is their perception and experience? Supportive Care in Cancer, 26, 1471–1478. 29168034 10.1007/s00520-017-3977-0

[CIT0036] Hong, Q. N., Pluye, P., Bujold, M. and Wassef, M. (2017) Convergent and sequential synthesis designs: implications for conducting and reporting systematic reviews of qualitative and quantitative evidence. Systematic Reviews, 6, 61. 28335799 10.1186/s13643-017-0454-2 PMC5364694

[CIT0037] Hooper, P., Jutai, J. W., Strong, G. and Russell-Minda, E. (2008) Age-related macular degeneration and low-vision rehabilitation: a systematic review. Canadian Journal of Ophthalmology, 43, 180–187. 18347620 10.3129/i08-001

[CIT0038] Hulvat, M. C. (2020) Cancer incidence and trends. Surgical Clinics of North America, 100, 469–481. 32402294 10.1016/j.suc.2020.01.002

[CIT0039] Hwang, P. W. and Braun, K. L. (2015) The effectiveness of dance interventions to improve older adults’ health: a systematic literature review. Alternative Therapy Health Medicine, 21, 64–70. PMC5491389 26393993

[CIT0040] Jenkins, E. and Wakeling, K. (2020) ‘You move as you feel and you feel as you move’: the practice and outcomes of a creative dance project for women living with or beyond cancer. Perspectives in Public Health, 140, 249–251. 32933433 10.1177/1757913920916778

[CIT0041] Kaltsatou, A., Mameletzi, D. and Douka, S. (2011) Physical and psychological benefits of a 24-week traditional dance program in breast cancer survivors. Journal of Bodywork & Movement Therapies, 15, 162–167. 21419356 10.1016/j.jbmt.2010.03.002

[CIT0042] Karathanou, I., Bebetsos, E., Filippou, F., Psirri, A. and Konas, I. (2020) Greek traditional dance as a practice for managing stress and anxiety in cancer patients. Journal of Cancer Education, 36, 1269–1276. 10.1007/s13187-020-01761-x 32388774

[CIT0043] Kasper, K. (2019) Sports training principles. Current Sports Medicine Reports, 18, 95–96. 30969230 10.1249/JSR.0000000000000576

[CIT0044] Koch, S. C., Riege, R., Tisborn, K., Biondo, J., Martin, L. and Beelmann, A. (2019) Effects of dance movement therapy and dance on health-related psychological outcomes. A meta-analysis update. Frontiers in Psychology, 10, 1806. 31481910 10.3389/fpsyg.2019.01806 PMC6710484

[CIT0047] Leite, B., de Bem Fretta, T., Boing, L. and Coutinho de Azevedo Guimaraes, A. (2021) Can belly dance and mat Pilates be effective for range of motion, self-esteem, and depressive symptoms of breast cancer women? Complementary Therapies in Clinical Practice, 45, 101483. 34517217 10.1016/j.ctcp.2021.101483

[CIT0048] Lizarondo, L., Stern, C., Carrier, J., Godfrey, C., Rieger, K., Salmond, S. et al. (2022) Chapter 8: Mixed methods systematic reviews. JBI Manual for Evidence Synthesis, 10.46658/JBIMES-20-09 (last accessed 30 November 2022). 32813460

[CIT0049] Loo, L. W. M., Nishibun, K., Welsh, L., Makolo, T., Chong, C. D., Pagano, I. et al. (2019) Using a cultural dance program to increase sustainable physical activity for breast cancer survivors – a pilot study. Complementary Therapies in Medicine, 47, 102197. 31780003 10.1016/j.ctim.2019.102197 PMC6905195

[CIT0050] Lopez, P., Galvao, D. A., Taaffe, D. R., Newton, R. U., Souza, G., Trajano, G. S. et al. (2021) Resistance training in breast cancer patients undergoing primary treatment: a systematic review and meta-regression of exercise dosage. Breast Cancer, 28, 16–24. 32815096 10.1007/s12282-020-01147-3

[CIT0051] Mishra, S. I., Scherer, R. W., Geigle, P. M., Berlanstein, D. R., Topaloglu, O., Gotay, C. C. et al. (2012) Exercise interventions on health-related quality of life for cancer survivors. Cochrane Database of Systematic Reviews, 8, CD007566. 10.1002/14651858.CD007566.pub2 PMC7387117 22895961

[CIT0052] Moher, D., Liberati, A., Tetzlaff, J. and Altman, D. G.; the PRISMA Group. (2009) Preferred reporting items for systematic reviews and meta-analyses: the PRISMA statement. Public Library of Science Medicine, 6. PMC3090117 21603045

[CIT0053] Molinaro, J., Kleinfeld, M. and Lebed, S. (1986) Physical therapy and dance in the surgical management of breast cancer. Physical Therapy, 66, 967–969. 3714815 10.1093/ptj/66.6.967

[CIT0054] National Cancer Registry Ireland. (2022) *Cancer in Ireland 1994-2020 Annual Statistical Report 2022 of the National Cancer Registry*. National Cancer Registry Ireland. https://www.ncri.ie/sites/ncri/files/pubs/NCRI_Annual%20Report2019_03102019.pdf (last accessed 29 November 2022).

[CIT0055] O’Higgins, C. M., Brady, B., O’Connor, B., Walsh, D. and Reilly, R. B. (2018) The pathophysiology of cancer-related fatigue: current controversies. Supportive Care in Cancer, 26, 3353–3364. 29961146 10.1007/s00520-018-4318-7

[CIT0056] Ormel, H. L., van der Schoot, G. G. F., Sluiter, W. J., Jalving, M., Gietema, J. A. and Walenkamp, A. M. E. (2018) Predictors of adherence to exercise interventions during and after cancer treatment: a systematic review. Psycho-Oncology, 27, 713–724. 29247584 10.1002/pon.4612 PMC5887924

[CIT0057] Østerås, H., Paulsberg, F. and Gravare Silbernagel, K. (2018) Are randomized control trials best for evaluating the effect of complex physical therapy interventions? British Journal of Sports Medicine, 52, 950–951. 29298752 10.1136/bjsports-2017-098456

[CIT0058] Park, S., Eo, W. and Lee, S. (2016) The relationship between health-related quality of life and survival in metastatic colorectal cancer patients treated with Korean medicine. Integrative Cancer Therapies, 17, 65–72. 28024424 10.1177/1534735416684015 PMC5950943

[CIT0059] Pinto-Carral, A., Molina, A. J., de Pedro, A. and Ayan, C. (2018) Pilates for women with breast cancer: a systematic review and meta-analysis. Complementary Therapies in Medicine, 41, 130–140. 30477829 10.1016/j.ctim.2018.09.011

[CIT0060] Piraux, E., Caty, G., Nana, F. A. and Reychler, G. (2020) Effects of exercise therapy in cancer patients undergoing radiotherapy treatment: a narrative review. SAGE Open Med, 8, 1–21. 10.1177/2050312120922657 PMC7301662 32595968

[CIT0061] Pisu, M., Demark-Wahnefried, W., Kenzik, K. M., Oster, R. A., Lin, C. P., Manne, S. et al. (2017) A dance intervention for cancer survivors and their partners (RHYTHM). Journal of Cancer Survivorship, 11, 350–359. 28070770 10.1007/s11764-016-0593-9 PMC5419878

[CIT0062] Raorane, N. S., Rao, K., Bhalerao, S., Redij, S., Kamthe, R., Gattani, S. et al. (2019) Effect of hula hoop on core muscle strength. International Journal of Applied Research, 3, 578–581.

[CIT0064] Roberson, D. N. and Pelclová, J. (2014) Social dancing and older adults: playground for physical activity. Ageing International, 39, 124–143.

[CIT0065] Rodrigues-Krause, J., Farinha, J. B., Ramis, T. R., Macedo, R. C. O., Boeno, F. P., dos Santos, G. C. et al. (2018) Effects of dancing compared to walking on cardiovascular risk and functional capacity of older women: a randomized controlled trial. Experimental Gerontology, 114, 67–77. 30389581 10.1016/j.exger.2018.10.015

[CIT0066] Rudolph, I., Schmidt, T., Wozniak, T., Kubin, T., Rütters, D. and Huebner, J. (2018) Ballroom dancing as physical activity for patients with cancer: a systematic review and report of a pilot project. Journal of Cancer Research and Clinical Oncology, 144, 759–770. 29423728 10.1007/s00432-018-2606-8 PMC11813434

[CIT0067] Schmidt, T., Rudolph, I., Wozniak, T., Ruetters, D., Van Mackelenbergh, M. T. and Huebner, J. (2018) Effect of ballroom dancing on the well-being of cancer patients: report of a pilot project. Molecular and Clinical Oncology, 9, 342–346. 30112180 10.3892/mco.2018.1663 PMC6090572

[CIT0069] Silverman, S. R., Schertz, L. A., Yuen, H. K., Lowman, J. D. and Bickel, C. S. (2012) Systematic review of the methodological quality and outcome measures utilized in exercise interventions for adults with spinal cord injuries. Spinal Cord, 50, 718–727. 22777488 10.1038/sc.2012.78

[CIT0070] Singh, J. (2013) Critical appraisal skills programme. Journal of Pharmacology and Pharmacotherapeutics, 4, 76–77.

[CIT0071] Slade, S. C., Patel, S., Underwood, M. and Keating, J. L. (2014) What are patient beliefs and perceptions about exercise for nonspecific chronic low back pain? A systematic review of qualitative studies. The Clinical Journal of Pain, 30, 995–1005. 24300225 10.1097/AJP.0000000000000044

[CIT0072] Soltero, E. G., Larkey, L. K., Kim, W. S., Chavez, J. B. R. and Lee, R. E. (2022) Latin dance and Qigong/Tai Chi effects on physical activity and body composition in breast cancer survivors: a pilot study. Complementary Therapies in Clinical Practice, 47. 10.1016/j.ctcp.2022.101554 35257993

[CIT0073] Stein, K. D., Syrjala, K. L. and Andrykowski, M. A. (2008) Physical and psychological long-term and late effects of cancer. American Cancer Society Journals, 112, 2577–2592. 10.1002/cncr.23448 PMC7047657 18428205

[CIT0074] Stout, N. L., Baima, J., Swisher, A., Winters-Stone, K. M. and Welsh, J. (2017) A systematic review of exercise systematic reviews in the cancer literature (2005-2017). PM R, 9, 347–384. 9S2 10.1016/j.pmrj.2017.07.074 PMC5679711 28942909

[CIT0075] Sturm, I., Baak, J., Storek, B., Traore, A. and Thuss-Patience, P. (2014) Effect of dance on cancer-related fatigue and quality of life. Supportive Care in Cancer, 22, 2241–2249. 24671434 10.1007/s00520-014-2181-8

[CIT0076] Sundaresan, P., Sullivan, L., Pendlebury, S., Kirby, A., Rodger, A., Joseph, D. et al. (2015) Patients’ perceptions of health-related quality of life during and after adjuvant radiotherapy for T1N0M0 Breast Cancer. Clinical Oncology, 27, 9–15. 25267304 10.1016/j.clon.2014.09.007

[CIT0077] Szalai, M., Lévay, B., Szirmai, A., Papp, I., Prémusz, V. and Bódis, J. (2015) A clinical study to assess the efficacy of belly dancing as a tool for rehabilitation in female patients with malignancies. European Journal of Oncology Nursing, 19, 60–65. 25201130 10.1016/j.ejon.2014.07.009

[CIT0078] Szalai, M., Szirmai, A., Füge, K., Makai, A., Erdélyi, G., Prémusz, V. et al. (2017) Special aspects of social support: qualitative analysis of oncologic rehabilitation through a belly dancing peer support group. European Journal of Cancer Care, 26. 10.1111/ecc.12656 28194904

[CIT0079] Thieser, S., Dorfler, J., Rudolph, I., Wozniak, T., Schmidt, T. and Hubner, J. (2021) Influence of ballroom dancing on fatigue, body image, self‑efficacy, and endurance of cancer patients and their partners. Medical Oncology, 38, 15. 33507443 10.1007/s12032-021-01459-0 PMC7843482

[CIT0080] Ward, Z. J., Scott, A. M., Hricak, H., Abdel-Wahab, M., Paez, D., Lette, M. M. et al. (2020) Estimating the impact of treatment and imaging modalities on 5-year net survival of 11 cancers in 200 countries: a stimulation-based analysis. The Lancet Oncology, 21, 1077–1088. 32758462 10.1016/S1470-2045(20)30317-X PMC8020599

[CIT0081] World Health Organisation. (2022) *Physical Activity*. https://www.who.int/news-room/fact-sheets/detail/physical-activity (last accessed 20 Feb 2023).

[CIT0082] Zhu, G., Zhang, X., Wang, Y., Xiong, H., Zhao, Y. and Sun, F. (2016) Effects of exercise intervention in breast cancer survivors: a meta-analysis of 33 randomised controlled trials. OncoTargets and Therapy, 9, 2153–2168. 27110131 10.2147/OTT.S97864 PMC4835133

