# Supplementary material for: The effectiveness of community dance in people with cancer: a mixed-methods systematic review and meta-analysis
Source: Health Promot Int. 2023 Aug 3;38(4):daad077. doi: 10.1093/heapro/daad077 (PMC10400145; doi:10.1093/heapro/daad077)
Supplement: daad077_suppl_Supplementary_Material [file daad077_suppl_supplementary_material.docx]

**Supplementary Material**

[The effectiveness of community dance in people with cancer: A mixed-methods systematic review and meta-analysis]

This document contains the supplementary material for the above paper.

Section 1 contains the Downs and Black Critical Appraisal Table and the Critical Appraisal Skills Programme Checklist.

Section 2 contains the table on intervention dosage for each study.

Section 3 contains the table of results on our outcomes on interest.

Table of Contents

[1 Critical Appraisal 1](#_Toc120744484)

[1.1 Downs and Black Critical Appraisal Table 1](#_Toc120744485)

[1.2 Critical Appraisal Skills Programme Checklist 1](#_Toc120744486)

[2 Critical Appraisal 1](#_Toc120744484)

[2.1 Downs and Black Critical Appraisal Table 1](#_Toc120744485)

[2.2 Critical Appraisal Skills Programme Checklist](#_Toc120744486)

[3 Outcomes of interest results summary 2](#_Toc120744487)

[3.1 Functional capacity 2](#_Toc120744488)

[3.2 Fatigue 3](#_Toc120744491)

[3.3 Quality of life 4](#_Toc120744492)

[3.4 Psychological 6](#_Toc120744493)

[3.5 Relationships 8](#_Toc120744494)

[3.6 Body image 8](#_Toc120744495)

[3.7 Physical activity levels 9](#_Toc120744496)

# Critical Appraisal

## Downs and Black Critical Appraisal Table

This supplementary material document outlines the critical appraisal results of each individual study.

|  | He et al.  2022 | Leite et al.  2021 | Pisu et al.  2017 | Kaltsatou et al. 2011 | Karathanou et al. 2020 | Soltero et al. 2022 | Sturm et al.  2014 | Szalai et al.  2015 | Carminatti et al. 2019 | Boing et al.  2018 | Hiansdt et al. 2021 | Loo et al.  2019 | Thieser et al. 2021 | Schmidt et al. 2018 | Molinaro et al. 1986 |
| --- | --- | --- | --- | --- | --- | --- | --- | --- | --- | --- | --- | --- | --- | --- | --- |
| **Q1** | 1 | 1 | 1 | 1 | 1 | 1 | 1 | 1 | 1 | 1 | 1 | 1 | 1 | 1 | 1 |
| **Q2** | 1 | 1 | 1 | 1 | 1 | 1 | 1 | 1 | 1 | 1 | 1 | 1 | 1 | 1 | 1 |
| **Q3** | 1 | 1 | 1 | 1 | 1 | 1 | 1 | 1 | 1 | 1 | 1 | 1 | 1 | 1 | 1 |
| **Q4** | 1 | 1 | 1 | 1 | 1 | 1 | 1 | 1 | 1 | 1 | 1 | 1 | 1 | 1 | 1 |
| **Q5** | 2 | 2 | 2 | 0 | 1 | 2 | 1 | 2 | 1 | 1 | 2 | 0 | 2 | 0 | 0 |
| **Q6** | 1 | 1 | 1 | 1 | 1 | 1 | 1 | 1 | 1 | 1 | 1 | 1 | 0 | 1 | 0 |
| **Q7** | 1 | 1 | 1 | 1 | 1 | 1 | 1 | 1 | 1 | 1 | 1 | 0 | 0 | 0 | 0 |
| **Q8** | 1 | 1 | 0 | 0 | 0 | 0 | 1 | 0 | 1 | 0 | 1 | 0 | 0 | 1 | 0 |
| **Q9** | 1 | 1 | 1 | 0 | 0 | 0 | 1 | 1 | 1 | 1 | 1 | 0 | 0 | 0 | 0 |
| **Q10** | 1 | 1 | 1 | 1 | 1 | 1 | 1 | 1 | 1 | 1 | 1 | 1 | 1 | 0 | 0 |
| **Q11** | 0 | 1 | 1 | 1 | 1 | 0 | 1 | 1 | 1 | 1 | 0 | 1 | 0 | 1 | 1 |
| **Q12** | 1 | 1 | 1 | 0 | 0 | 1 | 1 | 0 | 1 | 0 | 0 | 1 | 0 | 0 | 0 |
| **Q13** | 1 | 0 | 1 | 1 | 1 | 1 | 1 | 1 | 0 | 1 | 0 | 1 | 0 | 1 | 1 |
| **Q14** | 0 | 0 | 0 | 0 | 0 | 0 | 0 | 0 | 0 | 0 | 0 | 0 | 0 | 0 | 0 |
| **Q15** | 1 | 1 | 1 | 0 | 0 | 0 | 0 | 0 | 0 | 0 | 0 | 0 | 0 | 0 | 0 |
| **Q16** | 1 | 1 | 1 | 1 | 1 | 1 | 1 | 1 | 1 | 1 | 1 | 1 | 0 | 1 | 1 |
| **Q17** | 1 | 1 | 0 | 1 | 1 | 1 | 1 | 1 | 1 | 1 | 1 | 1 | 0 | 1 | 0 |
| **Q18** | 1 | 1 | 1 | 1 | 1 | 1 | 1 | 1 | 1 | 1 | 1 | 1 | 1 | 0 | 0 |
| **Q19** | 1 | 1 | 1 | 1 | 1 | 0 | 1 | 1 | 1 | 0 | 0 | 1 | 1 | 0 | 0 |
| **Q20** | 1 | 1 | 1 | 1 | 1 | 0 | 1 | 1 | 1 | 1 | 1 | 1 | 1 | 1 | 0 |
| **Q21** | 1 | 1 | 1 | 1 | 1 | 1 | 1 | 1 | 1 | 1 | 1 | 0 | 1 | 0 | 0 |
| **Q22** | 1 | 1 | 1 | 1 | 1 | 1 | 1 | 1 | 1 | 1 | 1 | 0 | 0 | 0 | 0 |
| **Q23** | 1 | 1 | 1 | 1 | 1 | 1 | 0 | 0 | 0 | 0 | 0 | 0 | 0 | 0 | 0 |
| **Q24** | 1 | 0 | 1 | 1 | 0 | 0 | 0 | 0 | 0 | 0 | 0 | 0 | 0 | 0 | 0 |
| **Q25** | 1 | 1 | 0 | 1 | 1 | 0 | 1 | 1 | 0 | 0 | 1 | 0 | 0 | 0 | 0 |
| **Q26** | 1 | 1 | 1 | 0 | 0 | 1 | 1 | 1 | 0 | 1 | 1 | 1 | 1 | 0 | 0 |
| **Q27** | 1 | 1 | 0 | 0 | 0 | 0 | 1 | 0 | 0 | 1 | 0 | 0 | 0 | 0 | 0 |
| **Total** | 26 | 25 | 23 | 19 | 19 | 18 | 23 | 21 | 19 | 19 | 19 | 15 | 12 | 11 | 7 |
| **Rating** | E | G | G | F | F | F | G | G | F | F | F | F | P | P | P |

E=Excellent, G=Good, F=Fair

## Critical Appraisal Skills Programme Checklist

|  | **Section A:**  **Validity** | | | | | | **Section B:**  **Results** | | | **Section C:**  **Local Impact** | **Total** |
| --- | --- | --- | --- | --- | --- | --- | --- | --- | --- | --- | --- |
|  | Q1 | Q2 | Q3 | Q4 | Q5 | Q6 | Q7 | Q8 | Q9 | Q10 |  |
| **Butler et al. 2016 [47]** | yes | yes | yes | yes | yes | no | yes | yes | yes | no | 8 |
| **Szalai et al. 2017 [48]** | yes | yes | yes | no | yes | no | yes | yes | yes | yes | 8 |
| **Jenkins & Wakeling 2020** | yes | no | yes | no | no | no | yes | no | no | no | 3 |

# Exercise prescription summary

This supplementary material document provides the information on exercise prescription in each individual study.

## Exercise prescription

| **Study** | **Intervention** | | | | | | | **Control** |
| --- | --- | --- | --- | --- | --- | --- | --- | --- |
|  | **Frequency**  **(times per week)** | **Intensity** | **Time (mins)** | **Type** | **Dosage per week (mins)** | **Study Length**  **(weeks)** | **Total Dosage Per Intervention (hours)** |  |
| Boing et al. 2018** | 2 | - | 60 | Belly dance | 120 | 12 | 24 | Maintain usual PA |
| Butler et al. 2016 [53] | 1 | - | - | Dance | - | 10 | - | No control group |
| Carminatti et al. 2019** | 2 | - | 60 | Belly dance | 120 | 12 | 24 | Maintain usual PA |
| He at al. 2022 | 5 | - | 30 | Chinese square set dance | 150 | 16 | 40 | Weekly health consultation sessions |
| Hiansdt et al. 2021 | 2 | moderate | 60 | Various dance | 120 | 12 | 24 | Maintain usual PA |
| Jenkins & Wakeling 2021 [55] | - | - | - | Various dance | - | 12 | - | No control group |
| Kaltsatou et al. 2011 | 3 | 60-85% max HR | 60 | Greek dance | 180 | 27 | 81 | Maintain usual PA |
| Karanthanou et al. 2020 | 2 | Low, then moderate, <120 bpm | 60 | Greek dance | 120 | 8 | 16 | Maintain usual PA |
| Leite et al. 2021 | 3 | moderate | 60 | Belly dance | 180 | 16 | 48 | 3 arm – 1. Pilates or 2. Educational activities and maintain PA |
| Loo et al. 2019 | 2 | Moderate, 50-70% max HR | 60 | Hula dance | 120 | 24 | 48 | No control group |
| Molinaro et al. 1986 [52] | 2 | - | 60 | Jazz and ballet | 120 | - | - | No control group |
| Pisu et al. 2017 | 1 | - | 45 | Ballroom dance | 45 | 14.9 | 11.175 | Maintain usual PA |
| Schmidt et al. 2018 [51] | 1 | - | 90 | Ballroom dance | 90 | unclear | - | No control group |
| Soltero et al. 2022 | 2 | - | - | Latin dance | - | 8 | - | Tai Chi |
| Sturm et al. 2014 | 2 | - | 60 | Dance | 120 | 5 | 10 | Standard care, counselling, achieve 120-180 mins of PA per week |
| Szalai et al. 2015*** | 1 | - | 90 | Belly dance | 90 | 52 | 78 | Standard medical care |
| Szalai et al. 2017 [54]*** | 1 | - | 90 | Belly dance | 90 | 52 | 78 | Standard medical care |
| Thieser et al. 2021 | 1 | - | 90 | Ballroom dance – standard and latin | 90 | 45 | 67.5 | No control group |

Abbreviations: bpm – beats per minute; HR – heart rate, PA – Physical Activity, **paper with same population – Carminatti & Boing, *** paper with same population – Szalai & Szalai.

_ - high dosage (>150 mins per week), _ - moderate dosage (100-150 mins per week), _- low dosage (<100 mins per week)

# Outcomes of interest results summary

This supplementary material document provides the individual study results for the outcomes of interest.

## Functional capacity

| **Study** | **Intervention Type** | **Outcome Measure** | **Within group difference T1-T0** | | **Between group differences  at T1** | |
| --- | --- | --- | --- | --- | --- | --- |
|  |  |  | **Mean difference** | **p-value** | **Overall outcome** | **p-value** |
| Kaltsatou et al. 2011 | Greek | 6 Minute Walk Test** | ↑ in dance (MD: 41.67)  No ↑ in control (MD: -15.38) | p> 0.05 | Greater ↑ in dance than control | n.r. |
| Pisu et al. 2017 | Ballroom with partner | 6 Minute Walk Test** | ↑ in dance (MD: 36.2)  ↑ in control (MD: 24.5) | **p= 0.03***  p= 0.06 | No difference between the groups | p= 0.54 |
| Sturm et al. 2014 | Dance | 6 Minute Walk Test** | ↑ in dance (MD: 53.51)  ↑ in control (MD: 9.5) | **p= 0.0005***  p= 0.411 | Greater ↑ in dance than control | **p= 0.008*** |
| Thieser et al. 2021 | Dance | 6 Minute Walk Test | n.r. | **p= 0.000** | ~ | ~ |

Abbreviations:

↑ - improvement; n.r. – not reported; ~ - no value due to no control group; *significantly different to control (p<0.05);

** included in meta-analysis; MD – Mean Difference (post – pre); T1 – post intervention; T0 – pre intervention (baseline)

Within group difference T1-T0 : ‘dance group pre intervention vs dance group post intervention’ ‘control group pre intervention vs control group post intervention’

Between group differences at T1: ‘dance group post intervention vs control group post intervention’

## Fatigue

| **Study** | **Intervention Type** | **Outcome Measure** | **Within group difference T1-T0** | | **Between group differences  at T1** | |
| --- | --- | --- | --- | --- | --- | --- |
|  |  |  | **Mean difference** | **p-value** | **Overall outcome** | **p-value** |
| Boing et al. 2018 | Belly | Piper Fatigue Scale** | ↑ in dance (MD: -1)  No ↑ in control (MD: 1) | **p= 0.036***  p= 0.614 | Greater ↑ in dance than control | p= 0.196 |
| He et al. 2022 | Square Dance | Brief Fatigue Inventory** | No ↑ in dance (MD: 1.49)  No ↑ in control (MD: 1.94) | n.r | Greater ↑ in dance than control | **p< 0.001*** |
| Loo et al. 2019 | Hula | Brief Fatigue Inventory | ↑ in dance (MD: -0.1)  No control group | p= 0.88 | ~ | ~ |
| Loo et al. 2019 | Hula | The Profile of Mood States Short Form – Fatigue | ↑ in dance (MD: 0.5)  No control group | p= 0.68 | ~ | ~ |
| Loo et al. 2019 | Hula | The European Organization for Research and Treatment of Cancer Quality-of-Life Core Questionnaire – Fatigue | ↑ in dance (MD: -1.4)  No control group | p= 0.79 | ~ | ~ |
| Sturm et al. 2014 | Dance | Numerical Rating Scale – Fatigue | ↑ in dance (MD: -2.11)  No ↑ in control (MD: 0.05) | **p= 0.001***  p= 0.887 | Greater ↑ in dance than control | **p= 0.0001*** |
| Sturm et al. 2014 | Dance | Functional Assessment of Chronic Illness Therapy: Fatigue** | ↑ in dance (MD: 6.15)  ↑ in control (MD: 3.3) | **p= 0.003***  p= 0.13 | Greater ↑ in dance than control | p> 0.05 |

Abbreviations:

↑ - improvement; ~ - no value due to no control group; *significant; *significantly different to control (p<0.05);

** included in meta-analysis; MD – Mean Difference (post – pre); T1 – post intervention; T0 – pre intervention (baseline)

Within group difference T1-T0 : ‘dance group pre intervention vs dance group post intervention’ ‘control group pre intervention vs control group post intervention’

Between group differences at T1: ‘dance group post intervention vs control group post intervention’

## Quality of life

| **Study** | **Intervention Type** | **Outcome Measure** | **Within group difference T1-T0** | | **Between group differences  at T1** | |
| --- | --- | --- | --- | --- | --- | --- |
|  |  |  | **Mean difference** | **p-value** | **Overall outcome** | **p-value** |
| Boing et al. 2018 | Belly | The European Organization for Research and Treatment of Cancer – Functional scale** | ↑ in dance (MD: 21)  ↑ in control (MD: 3) | **P= 0.002***  p= 0.601 | Greater ↑ in dance than control | p= 0.762 |
| Boing et al. 2018 | Belly | The European Organization for Research and Treatment of Cancer – Breast Cancer – symptomatic scale | ↑ in dance (MD: -25)  ↑ in control (MD: -5) | **p= 0.001***  p= 0.313 | Greater ↑ in dance than control | p= 0.354 |
| He et al. 2022 | Square Dance | Functional assessment of cancer therapy – breast (FACT-B): total score** | No ↑ in dance (MD: -6.79)  No ↑ in control (MD: -10.49) | n.r | Greater ↑ in dance than control | **p= 0.049*** |
| Kaltsatou et al. 2011 | Greek | Life Satisfaction Inventory** | ↑ in dance (MD: 11.67)  ↑ in control (MD: 0.01) | **p< 0.05***  p> 0.05 | Greater ↑ in dance than control | n.r. |
| Loo et al. 2019 | Hula | The European Organization for Research and Treatment of Cancer Quality-of-Life Core Questionnaire – Global Health Status/QOL | ↑ in dance (MD: 7.3)  No control group | p= 0.15 | ~ | ~ |
| Pisu et al. 2017 | Ballroom with partner | Short Form 12 – Physical Component Summary | ↑ in dance (MD: 0.57)  ↑ in control (MD: 2.11) | p= 0.67  p= 0.17 | No greater ↑ in dance than control | p= 0.67 |
| Pisu et al. 2017 | Ballroom with partner | Short Form 12 – Mental Component Summary** | ↑ in dance (MD: 6.51)  ↑ in control (MD: 0.67) | **p= 0.01***  p= 0.64 | Greater ↑ in dance than control | **p= 0.04*** |
| Pisu et al. 2017 | Ballroom with partner | 36-Item Short Form Health Survey – General Health | ↑ in dance (MD: 6.15)  ↑ in control (MD: 2.25) | p= 0.21  p= 0.47 | Greater ↑ in dance than control | p= 0.13 |
| Schmidt et al. 2018 | Ballroom with partner | Visual Analog Scale | ↑ in dance (MD: 1.27)  No control group | n.r. | ~ | ~ |
| Sturm et al. 2014 | Dance | The European Organization for Research and Treatment of Cancer Quality-of-Life Core Questionnaire – Global Health | No ↑ in dance (MD: 0.45)  No control group | p> 0.05 | ~ | ~ |
| Szalai et al. 2015 | Belly | The European Organization for Research and Treatment of Cancer Quality-of-Life Core Questionnaire** | ↑ in dance (MD: -4.73)  ↑ in control (MD: -3.58) | p> 0.05  p> 0.05 | Greater ↑ in dance than control | **p= 0.001*** |
| Szalai et al. 2015 | Belly | Campbells overall life satisfaction | ↑ in dance (MD: 2.19)  No ↑ in control (MD: -3.37) | **p< 0.05***  **p< 0.05*** | Greater ↑ in dance than control | **p= 0.001*** |

Abbreviations:

↑ - improvement; n.r. – not reported; ~ - no value due to no control group; *significant; ** included in meta-analysis;

MD – Mean Difference (post – pre); T1 – post intervention; T0 – pre intervention (baseline)

Within group difference T1-T0 : ‘dance group pre intervention vs dance group post intervention’ ‘control group pre intervention vs control group post intervention’

Between group differences at T1: ‘dance group post intervention vs control group post intervention’

## Psychological

| DEPRESSION | | | | | | |
| --- | --- | --- | --- | --- | --- | --- |
| **Study** | **Intervention Type** | **Outcome Measure** | **Within group difference T1-T0** | | **Between group differences  at T1** | |
|  |  |  | **Mean difference** | **p-value** | **Overall outcome** | **p-value** |
| Boing et al. 2018 | Belly | Becks Depression Inventory** | ↑ in dance (MD: -8)  - in control (MD: 3) | **p= 0.002***  p= 0.162 | Greater ↑ in dance than control | p= 0.908 |
| He et al. 2022 | Square Dance | Patient Health Questionnaire Depression Scale (PHQ-9) | No ↑ in dance (MD: 3)  No ↑ in control (MD: 4) | n.r | Greater ↑ in dance than control | **p< 0.001*** |
| Kaltsatou et al. 2011 | Greek | Becks Depression Inventory** | ↑ in dance (MD: -19.87)  ↑ in control (MD: -11.08) | **P< 0.05***  **P< 0.05*** | Greater ↑ in dance than control | p> 0.05 |
| Leite et al. 2021 | Belly | Becks Depression Inventory** | ↑ in dance (MD: -2.4)  ↑ in control (MD: 0)  ↑ in pilates (MD: 2,6) | n.r | Greater ↑ in dance than control | p= 0.792 |
| Loo et al. 2019 | Hula | Center for Epidemiological Studies Depression (CESD) Total Score | ↑ in dance (MD: -3.4)  No control group | p= 0.17 | ~ | ~ |
| Loo et al. 2019 | Hula | The Profile of Mood States Short-Form - Depression/dejection | ↑ in dance (MD: 0.3)  No control group | p= 0.78 | ~ | ~ |
| STRESS | | | | | | |
| **Study** | **Intervention Type** | **Outcome Measure** | **Within group difference T1-T0** | | **Between group differences at T1** | |
|  |  |  | **Mean difference** | **p-value** | **Overall outcome** | **p-value** |
| Karathanou et al. 2020 | Greek | Depression Anxiety Stress Scale – Stress | ↑ in dance (MD: -13.69)  No ↑ in control (MD: 0.08) | **p< 0.001***  **p< 0.001*** | Greater ↑ in dance than control | n.r. |
| ANXIETY | | | | | | |
| **Study** | **Intervention Type** | **Outcome Measure** | **Within group difference T1-T0** | | **Between group differences at T1** | |
|  |  |  | **Mean difference** | **p-value** | **Overall outcome** | **p-value** |
| Karathanou et al. 2020 | Greek | Depression Anxiety Stress Scale – Anxiety | ↑ in dance (MD: -12.78)  ↑ in control (MD: -0.08) | **p< 0.001***  **p= 0.013*** | Greater ↑ in dance than control | n.r. |
| Loo et al. 2019 | Hula | The Profile of Mood States Short-Form - Tension/Anxiety | ↑ in dance (MD: 0.9)  No control group  ↑ at 104 weeks f/u | p= 0.27  p= 0.09 | ~ | ~ |

Abbreviations:

↑ - improvement; n.r. – not reported; ~ - no value due to no control group; *significant; f/u – follow up; ** included in meta-analysis;

MD – Mean Difference (post – pre); T1 – post intervention; T0 – pre intervention (baseline)

Within group difference T1-T0 : ‘dance group pre intervention vs dance group post intervention’ ‘control group pre intervention vs control group post intervention’

Between group differences at T1: ‘dance group post intervention vs control group post intervention’

## Relationships

| **Study** | **Intervention Type** | **Outcome Measure** | **Within group difference T1-T0** | | **Between group differences  at T1** | |
| --- | --- | --- | --- | --- | --- | --- |
|  |  |  | **Mean difference** | **p-value** | **Overall outcome** | **p-value** |
| Loo et al. 2019 | Hula | Social Constraints Scale – Most Important Person | - in dance (MD: 0)  No control group  *↑ at 104 weeks f/u | p= 0.88  p= 0.31 | ~ | ~ |
| Loo et al. 2019 | Hula | Social Constraints Scale – Other People | - in dance (MD: 0)  No control group  *↑ at 104 weeks f/u | p= 0.86  p= 0.23 | ~ | ~ |
| Pisu et al. 2017 | Ballroom with partner | Dyadic Trust Scale | ↑ in dance (MD: 4.54)  ↑ in control (MD: 1.81) | p= 0.23  p= 0.42 | Greater ↑ in dance than control | p= 0.795 |
| Pisu et al. 2017 | Ballroom with partner | Dyadic Agreement and Happiness | ↑ in dance (MD: 2.46)  ↑ in control (MD: 1.25) | p= 0.16  p= 0.36 | Greater ↑ in dance than control | p= 0.572 |
| Szalai et al. 2015 | Belly | Short Form k-14 Perceived Social Support | ↑ in dance (MD: 2.33)  No ↑ in control (MD: -3.53) | **p= 0.000***  **p= 0.000*** | Greater ↑ in dance than control | **p= 0.000*** |

Abbreviations:

↑ - improvement; ~ - no value due to no control group; *significant; f/u – follow up; MD – Mean Difference (post – pre);

T1 – post intervention; T0 – pre intervention (baseline)

Within group difference T1-T0 : ‘dance group pre intervention vs dance group post intervention’ ‘control group pre intervention vs control group post intervention’

Between group differences at T1: ‘dance group post intervention vs control group post intervention’

## Body image

| **Study** | **Intervention Type** | **Outcome Measure** | **Within group difference T1-T0** | | **Between group differences at T1** | |
| --- | --- | --- | --- | --- | --- | --- |
|  |  |  | **Mean difference** | **p-value** | **Overall outcome** | **p-value** |
| Boing et al. 2018 | Belly | The European Organization for Research and Treatment of Cancer - Breast Cancer - Body Image | ↑ in dance (MD: 25)  ↑ in control (MD: 9) | **p= 0.037***  p= 0.388 | Greater ↑ in dance than control | p= 0.526 |
| Carminatti et al. 2019 | Belly | Body Image After Breast Cancer Questionnaire – vulnerability | ↑ in dance (MD: -4)  No ↑ in control (MD: 3) | p= 0.197  p= 0.234 | Greater ↑ in dance than control | p= 0.528 |
| Carminatti et al. 2019 | Belly | Body Image After Breast Cancer Questionnaire – body stigma | ↑ in dance (MD: -7)  - in control (MD: 0) | **p= 0.017***  p= 0.835 | Greater ↑ in dance than control | p= 0.303 |
| Carminatti et al. 2019 | Belly | Body Image After Breast Cancer Questionnaire – transparency | ↑ in dance (MD: -4)  No ↑ in control (MD: 1) | **p= 0.021***  p= 0.670 | Greater ↑ in dance than control | p= 0.795 |
| Carminatti et al. 2019 | Belly | Body Image After Breast Cancer Questionnaire – body concerns | ↑ in dance (MD: -2)  No ↑ in control (MD: 2) | p= 0.377  p= 0.670 | Greater ↑ in dance than control | p= 0.572 |
| Carmina tti et al. 2019 | Belly | Body Image After Breast Cancer Questionnaire – arm problems | ↑ in dance (MD: -2)  No ↑ in control (MD: 0) | p= 0.179  p= 0.801 | Greater ↑ in dance than control | p= 0.961 |
| Carminatti et al. 2019 | Belly | Body Image After Breast Cancer Questionnaire – limitations | ↑ in dance (MD: -2)  ↑ in control (MD: -1) | p= 0.400  p= 0.532 | Greater ↑ in dance than control | p= 0.512 |
| Carminatti et al. 2019 | Belly | Rosenberg Self-Esteem Scale | ↑ in dance (MD: 3)  - in control (MD: 0) | p= 0.075  p= 0.071 | Greater ↑ in dance than control | p= 0.822 |
| Thieser et al. 2021 | Dance | Body Image Scale | n.r. | p= 0.156 | ~ | ~ |

Abbreviations:

↑ - improvement; *significant; MD – Mean Difference (post – pre); T1 – post intervention; T0 – pre intervention (baseline)

Within group difference T1-T0 : ‘dance group pre intervention vs dance group post intervention’ ‘control group pre intervention vs control group post intervention’

Between group differences at T1: ‘dance group post intervention vs control group post intervention’

## Physical activity levels

| **Study** | **Intervention Type** | **Outcome Measure** | **Within group difference T1-T0** | | **Between group differences  at T1** | |
| --- | --- | --- | --- | --- | --- | --- |
|  |  |  | **Mean difference** | **p-value** | **Overall outcome** | **p-value** |
| Boing et al. 2018 | Belly | International Physical Activity Questionnaire (-total exercise mins per day) | No ↑ in dance (MD: -6)  No ↑ in control (MD: -7) | p= 0.623  p= 0.459 | Greater ↑ in dance than control | p= 0.088 |
| Loo et al. 2019 | Hula | Godin Leisure Time Exercise Questionnaire – Total Leisure Activity Score | ↑ in dance (MD: 8.5)  No control group  ↑ at 52 weeks f/u | p= 0.28  **p< 0.05*** | ~ | ~ |
| Pisu et al. 2017 | Ballroom with partner | Godin Leisure Time Exercise Questionnaire – Total Leisure Activity Score | ↑ in dance (MD: 13.1)  No ↑ in control (MD: 3.53) | **p= 0.01***  p= 0.59 | Greater ↑ in dance than control | **P< 0.05*** |
| Soltero et al. 2022 | Latin | Steps | ↑ in dance (MD: 454.91)  ↑ in control (MD: 4675.01) | p= 0.98  p= 0.80 | No greater ↑ in dance than control | n.r. |

Abbreviations:

↑ - improvement; ~ - no value due to no control group; *significant; f/u – follow up; MD – Mean Difference (post – pre);
T1 – post intervention, T0 – pre intervention (baseline)

Within group difference T1-T0 : ‘dance group pre intervention vs dance group post intervention’ ‘control group pre intervention vs control group post intervention’

Between group differences at T1: ‘dance group post intervention vs control group post intervention’
